# FUNKI: interactive functional footprint-based analysis of omics data

**DOI:** 10.1093/bioinformatics/btac055

**Published:** 2022-02-04

**Authors:** Rosa Hernansaiz-Ballesteros, Christian H Holland, Aurelien Dugourd, Julio Saez-Rodriguez

**Affiliations:** Institute for Computational Biomedicine, Heidelberg University, Heidelberg University Hospital, Faculty of Medicine, Bioquant, Heidelberg 69120, Germany; Institute for Computational Biomedicine, Heidelberg University, Heidelberg University Hospital, Faculty of Medicine, Bioquant, Heidelberg 69120, Germany; Faculty of Biosciences, Heidelberg University, Heidelberg 69120, Germany; Institute for Computational Biomedicine, Heidelberg University, Heidelberg University Hospital, Faculty of Medicine, Bioquant, Heidelberg 69120, Germany; Faculty of Biosciences, Heidelberg University, Heidelberg 69120, Germany; Institute for Computational Biomedicine, Heidelberg University, Heidelberg University Hospital, Faculty of Medicine, Bioquant, Heidelberg 69120, Germany

## Abstract

**Motivation:**

Omics data are broadly used to get a snapshot of the molecular status of cells. In particular, changes in omics can be used to estimate the activity of pathways, transcription factors and kinases based on known regulated targets, that we call footprints. Then the molecular paths driving these activities can be estimated using causal reasoning on large signalling networks.

**Results:**

We have developed FUNKI, a FUNctional toolKIt for footprint analysis. It provides a user-friendly interface for an easy and fast analysis of transcriptomics, phosphoproteomics and metabolomics data, either from bulk or single-cell experiments. FUNKI also features different options to visualize the results and run post-analyses, and is mirrored as a scripted version in R.

**Availability and implementation:**

FUNKI is a free and open-source application built on R and Shiny, available at https://github.com/saezlab/ShinyFUNKI and https://saezlab.shinyapps.io/funki/.

**Supplementary information:**

Supplementary data are available at *Bioinformatics* online.

## 1 Introduction

Multiple methods are conceived to infer the activities of specific processes or molecules using the abundance of known targets from omic data (Supplementary Table S1). We call them footprint-based methods (Dugourd and Saez-Rodriguez, 2019), and we have developed such tools for transcription factor (TF) from transcripts of target genes (Garcia-Alonso *et al.*, 2019), kinases from phosphorylated sites (Wirbel *et al.*, 2018) and pathways from transcripts of downstream responsive genes (Schubert *et al.*, 2018). These activities can then be used to contextualize large signalling networks by identifying paths that can explain the changes in activities via reverse causal reasoning (Dugourd *et al.*, 2021; Liu *et al.*, 2019), and be further linked to changes observed at the level of metabolite abundances (Dugourd *et al.*, 2021; Liu *et al.*, 2019).

FUNKI (FUNctional analysis toolKIt) is an user-friendly interface developed in R (Team, 2020), and designed using Shiny (Chang *et al.*, 2020), to analyze omics data using footprint methods. This application provides an interface for the R implementations (Bioconductor packages) for the aforementioned tools. All methods run on bulk data, and we have shown that they can also be applied to single-cell transcriptomics (Holland *et al.*, 2020a), not only for humans but also for mouse samples (Holland *et al.*, 2020b).

## 2 Features

**Fig. 1.**
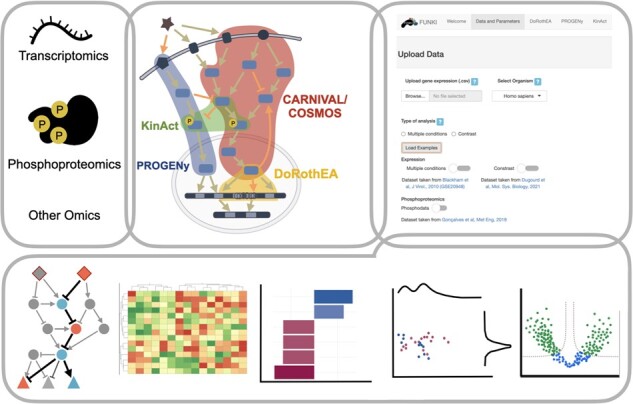
Graphical overview of analysis and visualization features provided by FUNKI. FUNKI provides a user interface to upload omics data, and then run DoRothEA, PROGENy, KinAct, CARNIVAL and COSMOS to estimate the activity of pathways, TFs and kinases. The results are visualized in diverse forms

The footprint methods implemented in FUNKI allow users to recover functional insight from several omics data without notions of programming. This application also enhances the analysis with an extended graphic visualization of the results. Thus, the typical FUNKI pipeline comprises three steps: (i) import the user’s omic data, (ii) select the analysis tool according to the data and question and (iii) visualize the results in tables and graphical representations (Fig. 1). 

Currently, the following tools are implemented:

### 2.1 DoRothEA

DoRothEA (Discriminant Regulon Expression Analysis) is a resource that links TFs with their downstream targets (Garcia-Alonso *et al.*, 2019). TF activities are computed from gene expression where the regulons (the collection of transcriptional targets for each TF) are the underlying gene sets.

### 2.2 PROGENy

PROGENy (Pathway RespOnsive GENes) is a footprint method developed to infer pathway activity from gene expression data (Schubert *et al.*, 2018). The scores are calculated using a linear model with weights based on consensus gene signatures obtained from publicly available perturbation experiments.

### 2.3 KinAct

KinAct estimates kinase activities based on abundance changes measures in target phosphorylation sites (Wirbel *et al.*, 2018) using the same algorithm as DoRothEA. Instead of TF-target interactions, KinAct uses collections of kinase–substrate interactions via OmniPath (Türei *et al.*, 2016) and phosphoproteomic data instead of transcriptomic data.

### 2.4 CARNIVAL and COSMOS

CARNIVAL (CAusal Reasoning for Network identification using Integer VALue programming) reconstructs signalling networks from downstream TF activities by finding the upstream regulators (Dugourd *et al.*, 2021; Liu *et al.*, 2019). COSMOS is an extension of CARNIVAL that provides a multiomic network to connect different types of omic data together, including transcriptomics, metabolomics and phosphoproteomics (Dugourd *et al.*, 2021; Liu *et al.*, 2019). Both methods identify coherent mechanistic hypotheses (subnetworks) that explain how the measured deregulation may be reached.

## 3 Implementation

FUNKI is a Shiny application developed using R programming language under version 4.0.2 and upgraded to run for 4.1.1 (Chang *et al.*, 2020; Team, 2020). It is directly accessible in the cloud through https://saezlab.shinyapps.io/funki/. The source code is freely available at https://github.com/saezlab/ShinyFUNKI, and it can be run locally in any platform (Windows, macOS and Linux) either downloading the repository or running it directly from GitHub (see https://saezlab.github.io/ShinyFUNKI/ for details).

## 4 Conclusion

FUNKI provides an intuitive user-friendly interface to run footprint methods from different omics. Together with the analysis implementation, FUNKI also incorporates several graphical representations to explore the results from different perspectives. Users with programming skills can take advantage of an extended script-based version of FUNKI for transcriptomic data (https://github.com/saezlab/transcriptutorial).

We plan to include further tools, such as oCEan, a method to explore unbalanced metabolic enzyme activity profiles (Sciacovelli *et al.*, 2021); and we also welcome contributions from other groups.

## Supplementary Material

btac055_Supplementary_Data
