# Supplementary material for: FUNKI: interactive functional footprint-based analysis of omics data
Source: Bioinformatics. 2022 Feb 4;38(7):2075–6. doi: 10.1093/bioinformatics/btac055 (PMC8963329; doi:10.1093/bioinformatics/btac055)
Supplement: btac055_Supplementary_Data [file btac055_supplementary_data.pdf]

Supplementary table 1

Name	Activity type	Available interface	Reference
Viper	TF/Kinase	No	(Alvarez *et al.*, 2016)
RoKAI	Kinase	No	(Yılmaz *et al.*, 2021)
KARP	Kinase	No	(Wilkes *et al.*, 2017)
KSEA	Kinase	No	(Hernandez-Armenta *et al.*, 2017)
KSTAR	Kinase	No	(Crowl *et al.*, 2021)
INKA	Kinase	No	(Beekhof *et al.*, 2019)
KEA3	Kinase	Yes	(Kuleshov *et al.*, 2021)
BART	TF	No	(Wang *et al.*, 2018)
TFEA.ChiP	TF	Yes	(Puente-Santamaria *et al.*, 2019)
oPOSSUM	TF	Yes	(Kwon *et al.*, 2012)
CHEA3	TF	Yes	(Keenan *et al.*, 2019)
MAGICACT	TF	No	(Roopra, 2018)
SPEED	Pathway	Yes	(Parikh *et al.*, 2010)
SPEED2	Pathway	Yes	(Rydenfelt *et al.*, 2020)

List of other methods to infer the activity of proteins from different omics based on the idea of looking at the target molecules (what we call the footprint-based approach). TF = Transcription Factor.

Bibliography

Alvarez,M.J. *et al.* (2016) Functional characterization of somatic mutations in cancer using network-based inference of protein activity. *Nat. Genet.*, **48**, 838–847.

Beekhof,R. *et al.* (2019) INKA, an integrative data analysis pipeline for phosphoproteomic inference of active kinases. *Mol. Syst. Biol.*, **15**, e8981.

Crowl,S. *et al.* (2021) KSTAR: An algorithm to predict patient-specific kinase activities from phosphoproteomic data. *BioRxiv*.

Hernandez-Armenta,C. *et al.* (2017) Benchmarking substrate-based kinase activity inference using phosphoproteomic data. *Bioinformatics*, **33**, 1845–1851.

Keenan,A.B. *et al.* (2019) ChEA3: transcription factor enrichment analysis by orthogonal omics integration. *Nucleic Acids Res.*, **47**, W212–W224.

Kuleshov,M.V. *et al.* (2021) KEA3: improved kinase enrichment analysis via data integration. *Nucleic Acids Res.*, **49**, W304–W316.

Kwon,A.T. *et al.* (2012) oPOSSUM-3: advanced analysis of regulatory motif over-representation across genes or ChIP-Seq datasets. *G3 (Bethesda)*, **2**, 987–1002.

Parikh,J.R. *et al.* (2010) Discovering causal signaling pathways through gene-expression patterns. *Nucleic Acids Res.*, **38**, W109-17.

Puente-Santamaria,L. *et al.* (2019) TFEA.ChIP: a tool kit for transcription factor binding site enrichment analysis capitalizing on ChIP-seq datasets. *Bioinformatics*, **35**, 5339–5340.

Roopra,A. (2018) MAGIC: A tool for predicting transcription factors and cofactors binding sites in gene sets using ENCODE data. *BioRxiv*.

Rydenfelt,M. *et al.* (2020) SPEED2: inferring upstream pathway activity from differential gene expression. *Nucleic Acids Res.*, **48**, W307–W312.

Wang,Z. *et al.* (2018) BART: a transcription factor prediction tool with query gene sets or epigenomic profiles. *Bioinformatics*, **34**, 2867–2869.

Wilkes,E.H. *et al.* (2017) Kinase activity ranking using phosphoproteomics data (KARP) quantifies the contribution of protein kinases to the regulation of cell viability. *Mol. Cell. Proteomics*, **16**, 1694–1704.

Yılmaz,S. *et al.* (2021) Robust inference of kinase activity using functional networks. *Nat. Commun.*, **12**, 1177.
